# The Role of M1 and M2 Macrophages in Prostate Cancer in relation to Extracapsular Tumor Extension and Biochemical Recurrence after Radical Prostatectomy

**DOI:** 10.1155/2014/486798

**Published:** 2014-03-11

**Authors:** M. Lanciotti, L. Masieri, M. R. Raspollini, A. Minervini, A. Mari, G. Comito, E. Giannoni, M. Carini, P. Chiarugi, S. Serni

**Affiliations:** ^1^Department of Urology, University of Florence, Careggi Hospital, Viale San Luca, 50134 Florence, Italy; ^2^Department of Pathology, University of Florence, Careggi Hospital, Florence, Italy; ^3^Department of Biomedical, Experimental and Clinical Sciences, University of Florence, Viale Morgagni 50, 50134 Florence, Italy

## Abstract

*Introduction*. The aim of our work was to investigate the causal connection between M1 and M2 macrophage phenotypes occurrence and prostate cancer, their correlation with tumor extension (ECE), and biochemical recurrence (BR). * Patient and Methods*. Clinical and pathological data were prospectively gathered from 93 patients treated with radical prostatectomy. Correlations of commonly used variables were evaluated with uni- and multivariate analysis. The relationship between M1 and M2 occurrence and BR was also assessed with Kaplan-Meier survival analysis. * Results*. Above all in 63.4% there was a M2 prevalence. M1 occurred more frequently in OC disease, while M2 was more represented in ECE. At univariate analysis biopsy and pathologic GS and M2 were statistically correlated with ECE. Only pathologic GS and M2 confirmed to be correlated with ECE. According to macrophage density BCR free survival curves presented a statistically significant difference. When we stratified our population for M1 and M2,we did not find any statistical difference among curves. At univariate analysis GS, pTNM, and positive margins resulted to be significant predictors of BCR, while M1 and M2 did not achieve the statistical significance. At multivariate analysis, only GS and pathologic stage were independent predictors of BR. * Conclusion*. In our study patients with higher density of M count were associated with poor prognosis; M2 phenotype was significantly associated with ECE.

## 1. Introduction

Several epidemiologic studies support the opinion that chronic inflammatory diseases are frequently associated with increased risk of various human cancers, even up to 25% of them [1, 2].

Prostate cancer (PCa) represents one of the most common cancers and the second leading cause of cancer-related death in men in the United States [3]. Actually there is a substantial epidemiological evidence that chronic inflammation is associated with PCa [4] and many studies aimed to investigate the causal connection between inflammation and PCa.

It has been recently observed that proliferative inflammatory atrophy (PIA) lesions are strictly related to chronic prostatic inflammation, and histological cellular transitions have been noted between areas of PIA and high-grade prostate intraepithelial neoplasia (HGPIN), and furthermore between PIA and PCa [5]. A key role of the PIA lesion is the presence of leukocyte infiltration, with the majority of cells belonging to the monocyte-macrophages lineage. Tumor-associated macrophages (TAMs) are a significant component of the inflammatory cell infiltrates in prostate cancer. Mononuclear cells and/or polymorphonuclear cells in both epithelial and stromal compartments promote carcinogenesis with their ability to communicate via a complex network of intercellular signalling pathways mediated by proinflammatory cytokines, their receptors, and cell surface adhesion molecules. TAMs may have both tumor stimulatory and/or -inhibitory properties, probably because they can, by mechanisms largely unknown, differentiate into either cytotoxic (M1) or tumor growth promoting (M2) states. In several murine cancer models including chemically and genetically induced primary lung tumors, prostate tumors, colon xenografts, and lung metastases, TAMs expressed M2 early during tumorigenesis [6].

The aim of our work was to investigate the causal connection between M1 and M2 phenotype macrophages occurrence with PCa and to evaluate their correlation with clinic-pathological commonly used variables and survival.

## 2. Material and Methods

In our tertially referral center we routinely store in a specific database clinical and pathological data of patient undergoing radical prostatectomy (RP). In order to study a greater amount of neoplastic tissue and to better locate prostate inflammation at the pathological examination, we decided to prospectively select 93 consecutive patients with stage cT2b-c PCa undergoing RP from January 2000 to December 2011. Clinical stage assessment was routinely made by digital rectal examination at the visit, transrectal ultrasound at the time of the biopsy, and endorectal coil magnetic resonance for evaluating local extension. CT scan and bone scintigraphy were required for patients with PSA ≥ 20 ng/mL and GS ≥ 7.

All patients received anterograde RP according to our previously published technique [7]. The follow-up schedule included serum PSA assay every 3 months for the first year, then every 6 months for the following 2 years, and yearly thereafter. Biochemical recurrence (BCR) was defined as evidence of PSA > 0.2 ng/mL on two consecutive measurements.

### 2.1. Statistical Analysis

Statistical analysis was performed using StatView software. Univariate analysis was carried out as follows: Student's *t*-test was used comparing continuous parametric variables, Mann Whitney test was used comparing continuous nonparametric variables, and Pearson chi square test was used comparing nominal variables.

The risk of ECE, related to the preoperative variables analyzed, was evaluated using the logistic regression model, and odds ratios and risk ratios were calculated.

In order to establish the correlation between macrophages phenotype and prognosis, the BCR-free survival rate was estimated by the Kaplan-Meier method. Statistical significance was verified by the log-rank test.

### 2.2. Tissue Specimens and Immunohistochemistry

All specimens were obtained from the archives of formalin-fixed, paraffin-embedded tissue blocks. Haematoxylin-eosin stained sections from each histological specimen were reevaluated to confirm the histological diagnosis of PCa, for the Gleason grade [8] for detecting perineural invasion, and for surgical margin status. All cases were also reevaluated regarding the World Health Organization (WHO) 2004 classification [9] and pathological *T* staging was performed [10]. In addition, a representative tissue block was selected for further analysis. The following immunohistochemical markers were evaluated: CD68 and CD163. The stains for CD68 and CD163 were considered positive when there was a strong granular cytoplasmic or cytoplasmic and membrane staining patterns in cells of monocyte/macrophages lineage.

### 2.3. Slide Grading

**Figure 1:**
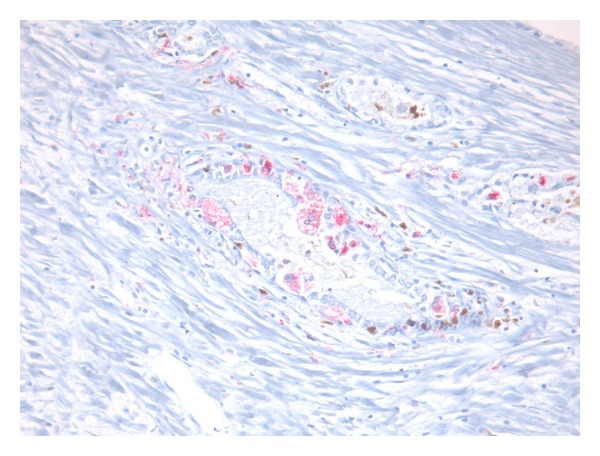
Presence of both M1 and M2 that were characterized by a red cytoplasm due to chromogen FAST RED for CD68 (M1) or with a brown cytoplasm due to chromogen diaminobenzidine (DAB) for CD163 (M2).

Macrophages were quantified by systematically screening the entire carcinoma area at low magnification using a 2,5x or 5x lens and selecting the areas with the highest density of macrophages and by counting them. M1 and M2 are distinguished by two different primary antibodies: anti-CD163 (for M2) and anti-CD68 (for M1) and two different chromogens: chromogen diaminobenzidine (DAB) for CD163 (M2, color brown) and chromogen FAST RED for CD68 (M1, color red). Therefore, we were able to recognize easily the two types of macrophages due to this double coloration (brown macrophages or M2 versus red macrophages or M1), and consequently we were able to count separately M1 and M2. We systematically screened the entire carcinoma area at low magnification, and in this way we selected the areas rich with macrophages (called: hot spot). After that, both M1 and M2 macrophages were manually counted at high magnification (Figure 1). Finally, the mean of both the number of M1 macrophages and M2 macrophages in these three hot spots was obtained. All counting was performed by one investigator (MRR) unaware of clinical data.

## 3. Results

Clinical and pathological characteristics of 93 patients included in our study are listed in Table 1.

Our patients presented median (IQR) preoperative PSA of 7.6 (1.01–86.8) and a prevalence of biopsy GS 6 (53.8%). Mean (SD) follow-up time after radical prostatectomy was 50.4 months (19.2). BCR occurred in 23 patients (24.7%) with a mean (SD) follow-up period of 26.3 (25.2) months. Among them 11 patients underwent delayed radiotherapy and 12 patients underwent palliative hormone therapy during follow-up period.

At the final anatomopathological evaluation 33 patients (35.5%) presented organ confined disease (OC), while in 60 pts (64.5%) there was ECE and positive surgical margins were found in 11 patients (11.8%). Patients with ECE presented higher prevalence of GS 7 to 8–10 and higher prevalence of PSA > 10 ng/mL with respect to the OC disease patients.

The macrophages prevalence is reported in Table 1 as follow: in 34 (36.6%) patients a higher prevalence of M1 was found, while in 59 (63.4%) patients there was a higher prevalence of M2. Mean (median) macrophage count of M1 and M2 in the three hot spots was 12.06 (6.0) and 17.18 (10.0), respectively.

M1 occurred more frequently in OC PCa, especially with GS 6 to 7 (mean number 18.6, median 11.6), while M2 resulted to be more represented in PCa with ECE and GS 7 to 8-10 (mean number 20.2, median 10).

When we correlated M1-M2 ratio to GS, biopsy cores, stage, and BCR at Student's *t*-test and Pearson *χ* ^2^ test, we found statistical correlation only with stage (*P* = 0.004). Moreover, at univariate analysis for ECE, pathological GS and M2 phenotype were statistically correlated with extracapsular extension (0.029, 0.0001, and 0.0079, resp.).

On the contrary, we did not find any statistical correlation between BCR and M1-M2 ratio, even if patients with higher prevalence of M1 phenotype presented better results.

At logistic regression analysis only specimen GS and M1-M2 ratio confirmed to be statistically correlated with ECE (*P* = 0.05, RR 10.65, and 95% CI 1.11–102.26 and *P* = 0.03, RR 0.295, and 95% CI 0.09–0.89, resp.) (see Table 2).

Moreover, at univariate analysis biopsy GS, pathological GS, pTNM, positive surgical margins, and high density of macrophages in three hot spots resulted to be independently predictive of BCR (*P* = 0.0009, 0.0006, and 0.0147, resp.), while substratification in M1 and M2 did not achieve the statistical significance. At Cox multivariable analysis only pathologic GS and stage resulted to be independent predictors of BCR (*r* = 19.146, *P* = 0.02 and *r* = 3.43, *P* = 0.05, resp.) (see Table 3).

**Figure 2:**
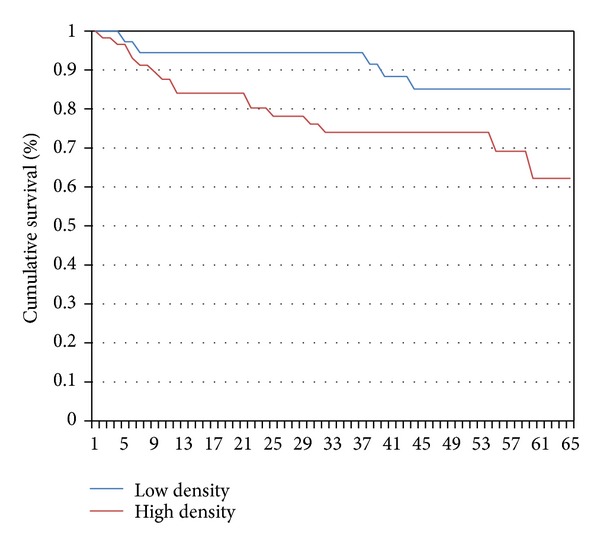
Biochemical recurrence (BCR) free survival curves at the Kaplan-Meier analysis of our 93 patients study population based on median number of macrophages. The blue line represents BCR free survival curve of patients with low density of macrophages M1 and M2 in three hot spots (n° of macrophages < median number), while the red line represents those with high density of M1 and M2 in three hot spots (>median number). BCR free survival rates at 36 and 60 months were 94.4 versus 74.0 and 85.1 versus 62.2, respectively, *P* = 0.05.

At the Kaplan-Meier survival analysis, the 36 and 60 months BR free survival rate for the global population resulted to be 84.6% and 72.5%, respectively. According to macrophage density, BCR free survival curves at the Kaplan-Meier analysis were 94.4 versus 74.0 and 85.1 versus 62.2 at 36 and 60 months, respectively, with a statistically significant difference among the curves (*P* = 0.05) (Figure 2).

**Figure 3:**
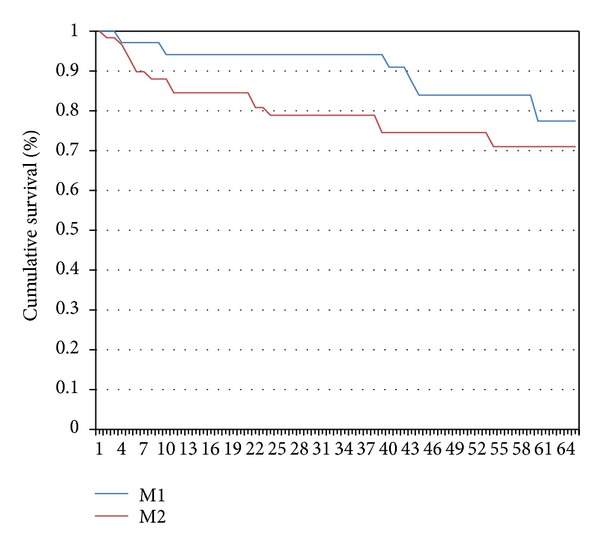
Biochemical recurrence (BCR) free survival curves at the Kaplan-Meier analysis of our 93 patients study population stratified for M1 and M2 macrophages prevalence. The blue line represents BCR free survival curve of patients with M1 macrophage prevalence, while the red line represents those with M2 macrophage prevalence. BCR free survival rates at 36 and 60 months were 94.1 versus 78.2 and 77.4 versus 71.0, respectively.

Moreover, when we stratified our patients for M1 and M2 macrophage phenotypes, we did not find any statistical difference among BCR free survival curves (log rank *P* = *ns*), although we observed that patients with prevalence of M2 macrophages showed a trend toward worst BCR free survival rates at 36 and 60 months compared to patients with M1 prevalence (78.2 versus 94.1 and 71.0 versus 77.4, resp.) (see Figure 3). When we analyzed survival curves for the category of patients with only ECE, among them, stratification for M1 and M2 macrophage phenotype did not allow us to establish a significant correlation with prognosis, although even in this instance patients with M2 phenotype prevalence confirmed to have a slightly worse prognosis.

## 4. Discussion

To date several clinicopathological factors have been reported as prognostic factors, but few studies have been reported on anticancer immune response by the host.

Prostate is constituted by epithelium and surrounding stroma, which itself consists of smooth muscle, extracellular matrix, and inflammatory cells. Inflammation has been thoroughly described as a key player in PCa, and among various inflammatory cell population, macrophages have been recognized as one of the major components. Chronic inflammation characterized by sustained tissue damage, damage induced cellular proliferation, and tissue repair have been analyzed in order to explain prostatic carcinogenesis, demonstrating a strong association between chronic prostatic inflammation, premalignant, and malignant changes in the prostatic epithelium in a prospective five years follow-up study on needle biopsy specimens [4]. Moreover, Nonomura et al. noticed that TAM infiltration was significantly correlated with serum PSA level, GS, or stage among the clinicopathological factors in prostate needle biopsy specimens [11].

Shimura et al. studied the association between TAM infiltration and disease-free survival after RP using whole mount sections, demonstrating that disease-free survival is significantly shorter for patients with a high level of TAMs than for those with a low level [12].

Macrophages are likely to encounter factors that most frequently polarise them toward M1 and M2 subtype macrophages, especially TAMs that express selected M2 protumoural functions, tumor progression, and metastasis [13].

In our study we observed that higher density of macrophage was statistically associated to poorer prognosis (*P* = 0.05, Figure 2). Moreover we found higher prevalence of M2 macrophage phenotype, which resulted more represented in PCa with ECE (*P* = 0.0079) and GS 7 to 8–10 and pT3a stage (*P* = *ns*). Our patients expressing more M2 phenotype frequently presented ECE and BCR, even if it was not confirmed at our statistical analysis. According to previous data published in the literature, TAMs generally exhibit an M2 phenotype known to promote angiogenesis, tumor growth, and metastasis [14]. To corroborate our preliminary results, in male mice TAM polarization in primary tumors at four distinct stages including PIN, well-differentiated, moderately differentiated, and poorly differentiated PCa has been examined [15].

In comparison to our global series of prostatectomies performed in our referral center as previously reported [7], we noticed in these 93 patient a slight higher incidence of extracapsular disease and GS 7. This was certainly due to our necessity to definitely locate macrophages in the prostate specimen at the final pathological evaluation. Indeed even in the literature high TAM count is statistically found in patients with higher stages (extracapsular extension) and grades (GS > 7) [11]. Even if it could be a limit of the study, our study population on the border between OC and ECE disease may be anyway extremely indicative for macrophage evaluation.

In addition to our preliminary results, further analysis on larger series of patients will allow us to better define M1-2 phenotype role in tumour aggressiveness and outcome.

## 5. Conclusion

It has become increasingly clear that TAMs are active players in the tumor aggressiveness. This is a preliminary study in which we laid down groundwork for further studies. In our study population with clinically localized PCa with stage cT2b-c, we found correlation between high macrophage infiltration and unfavorable items after RP. Moreover, M2 macrophage phenotype was significantly associated with extracapsular extension, even if this phenotype prevalence was not capable at the moment to predict BCR. Macrophage phenotype has demonstrated to be fascinating and valuable to rationalize a more aggressive adjuvant approach, even if further studies are needed to verify it.

## Figures and Tables

**Table 1:** Clinical presentation, pathologic findings, and follow-up of the 93 patients.

Macrophages population	Total of patients	M1	M2
*n* (%)	93	34 (36.6)	59 (63.4)
Count in three hot spots	Mean (median)	12.06 (6)	17.18 (10)
Density of macrophages M1 and M2 in three hot spots according to median number	43 below median number 50 above median number		

Preoperative variables			
Age (yy) median (IQR)	67 (45–75)	64 (55–74)	67 (45–75)
Total PSA (ng/mL) median (IQR)	7.6 (1.0–86.8)	8.3 (2.5–47.7)	7.0 (1–76.8)
Biopsy Gleason score *n* (%)			
6	50 (53.8)	19 (55.9)	31 (52.6)
7	28 (30.1)	12 (35.3)	16 (27.1)
8–10	15 (16.1)	3 (8.8)	12 (20.3)

Postoperative variables			
Organ confine disease (OC) *n* (%)	33 (35.5)	19 (55.9)	14 (23.8)
Extracapsular extension (ECE) *n* (%)	60 (64.5)	15 (44.1)	45 (76.2)
TNM stage *n* (%)			
T2	33 (35.5)	19 (55.9)	14 (23.8)
T3a	35 (37.6)	7 (20.6)	28 (47.4)
T3b	23 (24.8)	8 (23.5)	15 (25.4)
T4	2 (2.1)	0	2 (3.4)
Pathologic Gleason score *n* (%)			
6	30 (32.2)	13 (38.2)	17 (28.8)
7	40 (43)	16 (47.1)	24 (40.7)
8–10	23 (24.8)	5 (14.7)	18 (30.5)
Lymph node involvement *n* (%)	5 (5.3)	2 (5.9)	3 (5.1)
Positive surgical margin *n* (%)	11 (11.8)	2 (5.9)	9 (15.2)
Follow-up (months) mean (SD)	50.4 (19.2)	55.5 (21.2)	47.6 (16.3)
Biochemical recurrence *n* (%)	23 (24.7)	7 (20.6)	16 (27.1)

**Table 2:** Univariate (Pearson *χ* ^2^ test, *t*-test, and Mann Whitney test) and multivariate (logistic regression) analysis of common variables to predict extracapsular extension (ECE) of PCa.

Variables	Univariate analysis *P* value	Multivariate analysis		
		*P* value	Risk ratio	95% CI
Preoperative PSA	Ns	Not included in analysis		
Biopsy GS > 7	**0.029**	0.07	4.21	1.10–19.62
Anatomopathological GS > 7	**0.0001**	**0.05**	10.65	1.11–102.26
Lymphnode invasion	Ns	Not included in analysis		
M1/M2	**0.0079**	**0.03**	**0.295**	**0.09**–**0.89**

**Table 3:** Univariate (Pearson *χ* ^2^ test, Mann Whitney Test, and *t*-test) and multivariate (Cox proportional hazard model) analysis of common variables to predict BCR.

Variables	Univariate analysis *P* value	Multivariate analysis		
		*P* value	Risk ratio	95% CI
Preoperative PSA	Ns	—		
Biopsy GS > 7	**0.0009**	0.07	2.26	1.1–10.47
Anatomopathological GS > 7	**0.0006**	**0.02**	**16.04**	**1.44–177.9**
Pathological stage	**0.0147**	**0.03**	**3.43**	**1.09–11.8**
Lymphnode invasion	Ns	—		
Status	Ns	—		
Surgical margin status	Ns	—		
High density of macrophages M1 and M2 in three hot spots (above median value)	**0.05**	0.09	2.53	1.6–9.67
M1-2 phenotype	Ns	—		
